# Tularemia in Germany—A Re-emerging Zoonosis

**DOI:** 10.3389/fcimb.2018.00040

**Published:** 2018-02-16

**Authors:** Mirko Faber, Klaus Heuner, Daniela Jacob, Roland Grunow

**Affiliations:** ^1^Gastrointestinal Infections, Zoonoses and Tropical Infections (Division 35), Department for Infectious Disease Epidemiology, Robert Koch Institute, Berlin, Germany; ^2^Working Group, Cellular Interactions of Bacterial Pathogens, Centre for Biological Threats and Special Pathogens, Division 2 (ZBS 2), Robert Koch Institute, Berlin, Germany; ^3^Highly Pathogenic Microorganisms, Centre for Biological Threats and Special Pathogens, Division 2 (ZBS 2), Robert Koch Institute, Berlin, Germany

**Keywords:** *Francisella tularensis*, tularemia, epidemiology, case report, one health, Germany, ecology, veterinary medicine

## Abstract

Tularemia, also known as “rabbit fever,” is a zoonosis caused by the facultative intracellular, gram-negative bacterium *Francisella tularensis*. Infection occurs through contact with infected animals (often hares), arthropod vectors (such as ticks or deer flies), inhalation of contaminated dust or through contaminated food and water. In this review, we would like to provide an overview of the current epidemiological situation in Germany using published studies and case reports, an analysis of recent surveillance data and our own experience from the laboratory diagnostics, and investigation of cases. While in Germany tularemia is a rarely reported disease, there is evidence of recent re-emergence. We also describe some peculiarities that were observed in Germany, such as a broad genetic diversity, and a recently discovered new genus of *Francisella* and protracted or severe clinical courses of infections with the subspecies *holarctica*. Because tularemia is a zoonosis, we also touch upon the situation in the animal reservoir and one-health aspects of this disease. Apparently, many pieces of the puzzle need to be found and put into place before the complex interaction between wildlife, the environment and humans are fully understood. Funding for investigations into rare diseases is scarce. Therefore, combining efforts in several countries in the framework of international projects may be necessary to advance further our understanding of this serious but also scientifically interesting disease.

## Introduction

Germany represents a low incidence region with regard to tularemia in humans caused by *Francisella tularensis*. We highlight some peculiarities of the pathogen as observed in Germany and describe the epidemiology, outbreaks and possible sources of infection, different clinical courses and aspects of diagnosis as well as underline the one-health aspect of tularemia considering this disease relevant for human, animal, and environmental health. Tularemia in Germany is described as an example for many other European countries with a similar epidemiological situation, and parallels might be helpful to initiate further research on national and international levels.

## The pathogen

Tularemia is caused by infection with the facultative intracellular, gram-negative bacterium *Francisella tularensis* (Ellis et al., 2002; Sjöstedt, 2011). The most clinically relevant subspecies are *F. tularensis* ssp. *holarctica* distributed over the whole northern hemisphere and the more virulent ssp. *tularensis* which is almost exclusively found in North America and associated with lethal pulmonary infections (Ellis et al., 2002). Further species or subspecies of the genus *Francisella* have a low or unknown pathogenicity for humans (e.g., *F. tularensis* ssp. *novicida* and *F. tularensis* ssp. *mediasiatica* found in North America and Asia, respectively) (Ellis et al., 2002; Kingry and Petersen, 2014; Challacombe et al., 2017). Only recently, the presence of *F. tularensis* ssp. *holarctica* has been confirmed in the southern hemisphere (Eden et al., 2017).

In Germany, *F. tularensis* ssp. *holarctica* is the only subspecies known to cause disease in patients and animals. Only one other *Francisella* species, *Francisella* sp. isolate W12-1067 isolated from a water reservoir of a cooling tower of a hospital, has been found in Germany (Rydzewski et al., 2014). This isolate is ~89% identical to the chromosomal DNA of the published strain *F. guangzhouensis*, indicating that there may be additional, yet unidentified *Francisella* species present in Germany. Recently, a Chinese group proposed a strain related to *Francisella* sp. strain W12-1067 to represent a new genus called *Allofrancisella* (Qu et al., 2016). However, the identification of new *Francisella* species (Challacombe et al., 2017) and additional further research will reveal whether the proposal of a new genus can be confirmed and whether it is pathogenic for humans.

Studies on bacterial isolates from wild animals and humans revealed an unexpectedly high genetic diversity of *F. tularensis* ssp. *holarctica* in Germany (Gehringer et al., 2013; Müller et al., 2013; Schulze et al., 2016). In a recent study the phylogenetic analysis of isolates from wild animals from the Berlin/Brandenburg region revealed three new subclades within the phylogenetic tree, subclade B.71 from a raccoon dog [*Nyctereutes procyonoides*], subclade B.74 from a red fox [*Vulpes vulpes*] and subclade B.75 from an Eurasian beaver [*Castor fiber albicus*] (Schulze et al., 2016). The genetic diversity is not only of academic interest: In the phylogenetic analysis, erythromycin-susceptible *F. tularensis* ssp. *holarctica* cluster with biovar I and erythromycin-resistant with biovar II. While biovar I is mainly found in Western Europe, biovar II occurs in Northern and Eastern Europe (Kudelina, 1973; Ellis et al., 2002; Svensson et al., 2009a; Vogler et al., 2009a,b; Karlsson et al., 2016). In a recent study, 94 isolates were susceptible to erythromycin, which defines biovar I (genotypes B.4 and B.6), while 34 were resistant (biovar II; genotype B.12). Both *Francisella* biovars are present in Germany (Tomaso et al., 2017). It is still under debate whether strains of genotype B.6 may have a higher pathogenic potential than strains belonging to the B.12 genotype (Gyuranecz et al., 2010; Origgi and Pilo, 2016; Hestvik et al., 2017; Kreizinger et al., 2017). There are further publications describing the phylogeographic pattern of *Francisella* in Europe (Vogler et al., 2009a; Gyuranecz et al., 2012; Karlsson et al., 2013; Origgi et al., 2014; Thelaus et al., 2014; Dwibedi et al., 2016).

The high genetic diversity observed in *F. tularensis* isolates suggests that additional *Francisella* pheno genotypes and remain to be discovered. Germany might represent a “melting pot,” a region where, within the postulated spread of the pathogen from east to west, strains are mixed, re-assorted and give rise to further variants with still unknown characteristics (Jusatz, 1952a, 1961; Chanturia et al., 2011; Dwibedi et al., 2016). Phylogenetic studies also revealed a spread of tularemia from Scandinavia to the south of Europe (Karlsson et al., 2013; Dwibedi et al., 2016). More research on phylogenetic relations and pathogenicity of specific isolates is required for a better understanding of how genetics correlates with environmental habitats, reservoirs, vectors, and transmission routes.

## Occurrence of tularemia in germany–past and present

Historical publications describe the spread of tularemia from Eastern Europe to Western Europe, crossing the German geographical territory since the nineteenth century (Jusatz, 1952b). The cited early descriptions of tularemia assumed that the disease was already known under names such as “epidemic lymphadenitis,” “Plague-like lymphadenitis,” and “Influenza-like disease of water hole hunters.” A verification of such historic cases of lymphadenitis as tularemia would clarify the early occurrence and distribution of the disease in Europe.

More information is available for the time following the Second World War: a retrospective study revealed 687 cases reported from 1949 to 2005 (Grunow and Priebe, 2007), 515 of which were observed until 1959. This was attributed to the socio-economic situation after the war. The incidence of tularemia is known to increase during or after armed conflicts due to a decline in hygiene, housing conditions and food safety. This was also observed e.g., in Kosovo after the armed conflicts when two outbreaks of tularemia occurred in 2000–2002 with more than 500 confirmed cases in a region that had previously been likely to be non-endemic (Grunow et al., 2012). The suspected reason for these outbreaks was a strong increase in the population of small rodents due to crops left on the field. Beginning with the cold season, infected animals came in close contact with human dwellings and contaminated accessible food storages and unprotected drinking water sources. From 1960 to 2004, between 9 and 34 cases per 5-year intervals were reported in Germany, indicating a very low incidence or poor reporting activity despite the fact that tularemia was a notifiable disease in both East and West Germany during this period (Grunow and Priebe, 2007).

Today, tularemia is a notifiable disease in Germany according to the infection protection act of 2001. For surveillance purposes, a case is defined as a person with symptoms and laboratory confirmation of a recent *F. tularensis* infection, indicated by at least one of the four following methods: Antigen detection by e.g., enzyme immune assays or immunofluorescence assays; isolation of the living pathogen by cultivation; detection of specific nucleic acids, e.g., by polymerase chain reactions; or detection of specific antibodies with an increase of the titer in paired serum samples taken with a difference in time along the clinical course or one clearly high titer of antibodies (http://www.rki.de/DE/Content/Infekt/IfSG/Falldefinition/Downloads/Falldefinitionen_des_RKI.pdf?__blob=publicationFile; https://survstat.rki.de/default.aspx).

**Figure 1:**
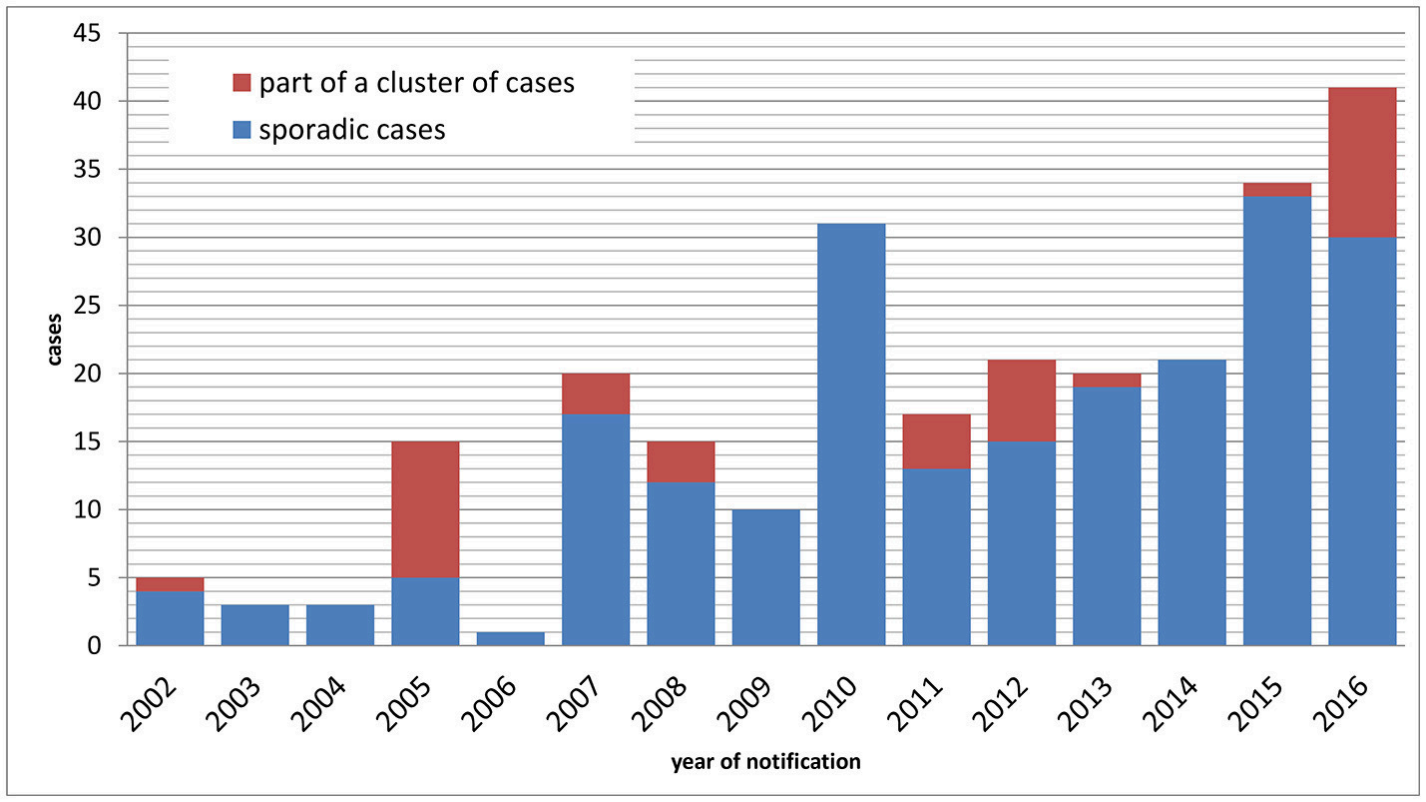
Notified cases of tularemia (sporadic and non-sporadic cases) by year of notification, Germany, 2002–2016.

Between 1 January 2002 and 31 December 2016, *n* = 257 cases of tularemia were notified in Germany (Figure 1), corresponding to a mean yearly incidence of 0.03 cases per 100,000 population (range: 0.00–0.05). In patients presenting with lymphadenitis and fever, tularemia is rarely considered as a differential diagnosis by clinicians and diagnostic laboratories, therefore it can be assumed that tularemia is subject to significant underdiagnosis and underreporting in Germany. Of the 257 cases reported, 217 were sporadic cases and 40 were part of case clusters. Age of the patients ranged from 1 to 87 years (mean: 46) and the male to female ratio was 2.06. While a median of three annual cases were notified from 2002 through 2006, a continuous increase with a maximum of 41 cases in 2016 was observed thereafter (Figure 1). It is unclear whether this increase is due to an actual increase in infection pressure and clinical cases or whether it is the result of increased awareness and more frequent testing. However, a relatively stable proportion of hospitalized cases suggests that the increase is not the result of a change in sensitivity of the surveillance system.

**Figure 2:**
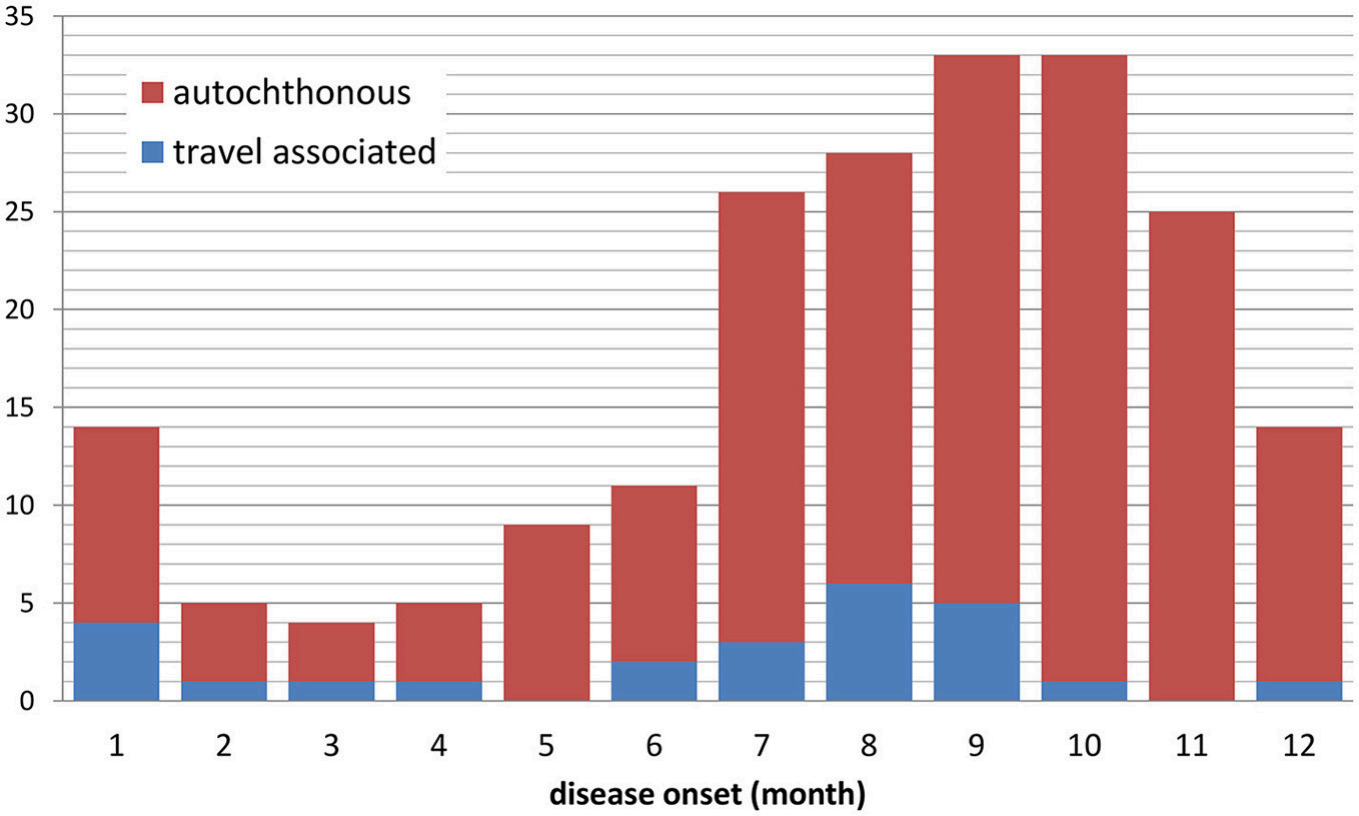
Notified cases of tularemia with and without travel history by month of disease onset, Germany, 2002–2016 (*n* = 207 with available information).

**Figure 3:**
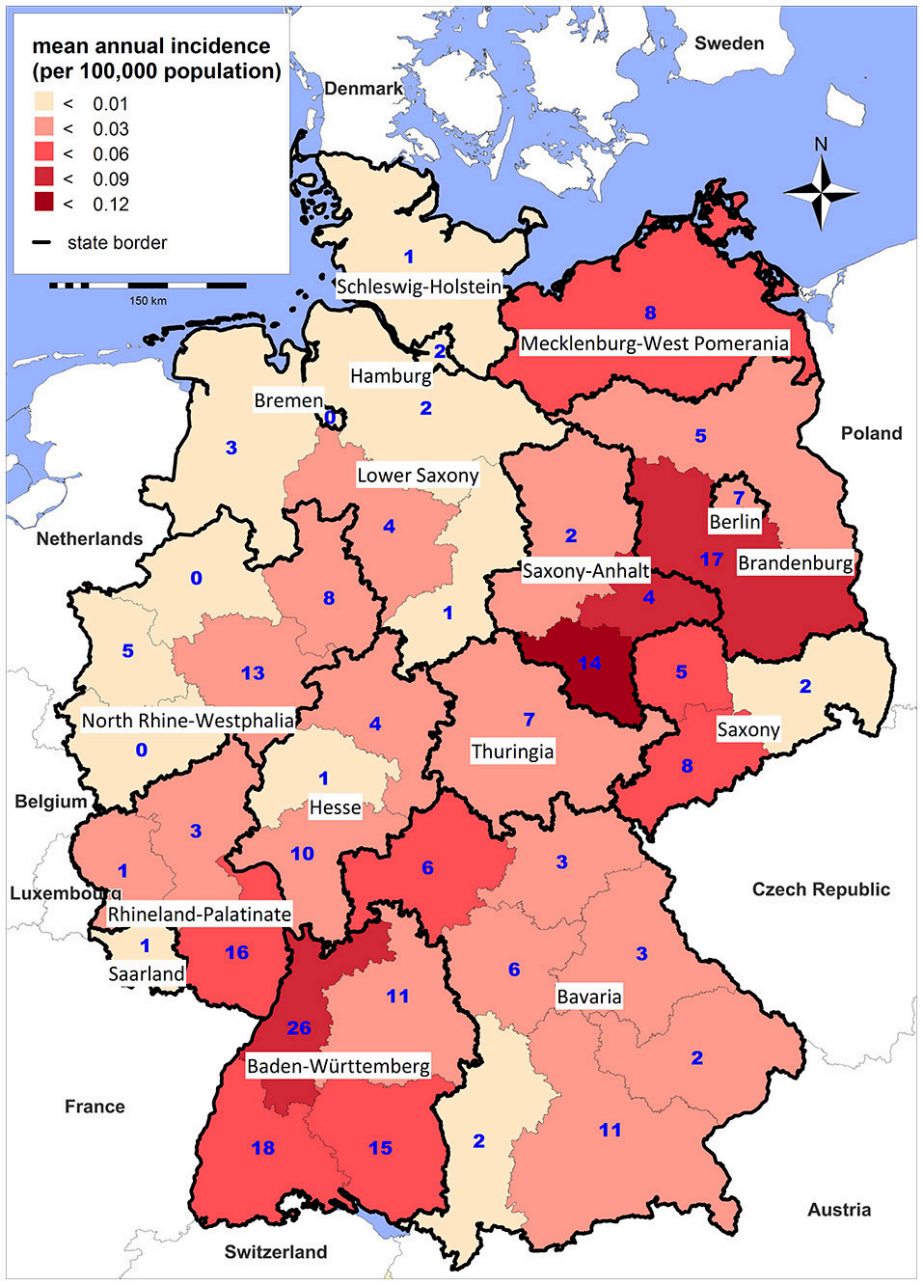
Mean annual incidence per 100,000 population (shading) and total number (blue digits) of notified tularemia cases by district, Germany 2002–2016.

Tularemia is a clearly seasonal disease in Germany with most patients (68%) reporting symptom onset from July through November (Figure 2) when reservoir animal populations are peaking and frequent outdoor activities (such as hunting, farming, fishing, hiking etc.) facilitate contact between wildlife and humans. This is in concordance with the seasonal occurrence of tularemia cases in Europe (Hestvik et al., 2015). Imported cases only account for a small fraction of the total case load (28 of 227, 12.5%), peaking after the summer holiday season in August and after Christmas/New Year. It is remarkable that cases until and after 2001 were reported from almost all Federal States of Germany. Between 2002 and 2016 the highest mean annual incidences were recorded in parts of Saxony-Anhalt, Baden-Württemberg and Brandenburg (Figure 3).

Although there is large variability in the regional incidence of tularemia, long-term surveillance data indicate that the pathogen can be found all-over Germany. The variation in the number of reported cases from the different Federal States could be explained by (i) variations in actual disease incidence as a result of varying exposure risks or infection pressure or (ii) variations in diagnostic consideration of tularemia and reporting activity due to differences in awareness of health care workers for the disease. Indeed, serological studies indicate that most infections are not diagnosed and reported in Germany (Al Dahouk et al., 2003; Jenzora et al., 2008; Kaysser et al., 2008; Splettstoesser et al., 2009; Gehringer et al., 2013; Kuehn et al., 2013; Müller et al., 2013; Otto et al., 2014). Two cross-sectional studies have shown a relatively high seroprevalence; one population-representative study from 2004 with 6,617 sera and one study conducted in a small town in Baden-Württemberg in 2009 with 2,416 sera revealed positive results in 0.23% and 2.3% of the sera, respectively (Porsch-Ozcürümez et al., 2004; Splettstoesser et al., 2009). Serological studies in hunters as a putative population at high risk have shown a seroprevalence of up to 1.7% (Jenzora et al., 2008).

It can be concluded that *Francisella tularensis* has been endemic for centuries on the territory today known as Germany and has a wide-spread distribution among wildlife. In humans tularemia probably represents a re-emerging disease with a high proportion of undiagnosed cases. Better awareness and knowledge of the disease among health care personnel is required for a timely diagnosis and treatment of cases. More research is required for a better understanding of the burden of disease and public health impact of tularemia in Germany.

## Outbreaks in humans and sources of infections

In Germany, outbreaks or clusters of tularemia occur rarely and are defined as at least two cases with an epidemiological link (such as a common source of exposure).

Between 2002 and 2016, 10 case clusters were reported. Except for two with a connection to situations in other countries and one large outbreak caused by grape must (unintentional contamination), the remaining eight clusters consisted of 2–10 cases and were associated with contact to wild animals, five of them in the context of hunting activities (Straube and Hess, 1986; Hofstetter et al., 2006; Schätzle and Schwenk, 2008; Hauri et al., 2010; Schubert et al., 2011; Kohlmann et al., 2014; Boone et al., 2015; Borde et al., 2017).

Five of the above clusters were reported in connection with consumption of or contact to infected hares. The largest one occurred in 2005 with a total of 10 affected hunters participating in a hare hunting event (Hofstetter et al., 2006; Hauri et al., 2010). For cases that were not part of clusters, tick bites have been suspected as the source of infection besides contact to hares (Lübbert et al., 2009; Boone et al., 2015; Borde et al., 2017). Indeed, prevalence studies in ticks in the south-west of Germany revealed presence of *Francisella* in 8% of 916 investigated *Ixodes ricinus* while *Dermacentor* species clustered with *Francisella* endosymbionts (Gehringer et al., 2013).

## Tularemia in animals

Tularemia case clusters in humans are often preceded by outbreaks in wildlife and detection of the pathogen in captured animals (Mörner, 1992; Splettstoesser et al., 2009); therefore much effort has been taken to describe the distribution of the pathogen in relevant reservoir animals. A serological study in various wildlife species in Brandenburg (the state surrounding the German capital Berlin), revealed a total of 101/1,353 positive sera (7.5%) in foxes (*Vulpes vulpes*), raccoon dogs (*Nyctereutes procyonoides*) and wild boar (*Sus scrofa*) (Kuehn et al., 2013). There is also serological evidence for *Francisella* infections in zoo animals: One seroconversion was documented in a hippo (*Hippopotamus amphibious*) between 2003 and 2004 (Kuehn et al., 2013). Other serological and bacteriological studies confirmed a high sero- and pathogen prevalence in wildlife including hares in Germany (Al Dahouk et al., 2003; Müller et al., 2013; Otto et al., 2014).

In a recent study, 3 out of 29 animals (10%) examined were *F. tularensis* positive which was in good agreement with the results mentioned above, and the presence of *F. tularensis* in raccoon dogs and red foxes could be confirmed (Schulze et al., 2016). Thus *F. tularensis* was isolated from animal species not previously reported as natural hosts in Germany. In the case of a beaver deceased from tularemia in Brandenburg, it could be confirmed that animals with high bacterial load may act as local amplifiers in Germany (Otto et al., 2015; Schulze et al., 2016). qPCR analyses indicated that *F. tularensis* persisted in the aquatic environment during one climatic season, but apparently no longer (Schulze et al., 2016).

In 2004, an outbreak of tularemia occurred in semi-free-living common marmosets (*Callithrix jacchus*) (Mätz-Rensing et al., 2007). *F. tularensis* was identified as the cause of a sudden die-off of 5 out of 62 animals. As all animals had been born at the facility, the outbreak was autochthonous meaning the source of infection was at the place where it occurred (Splettstoesser et al., 2007). A subsequent study in rodents in central Germany identified bank voles (*Myodes glareolus*), water voles (*Arvicola terrestris*), field voles (*Microtus agrestis*), common voles (*Microtus arvalis*) and yellow-necked field mice (*Apodemus flavicollis*) as potential sources of *Francisella* infections (Kaysser et al., 2008; Gyuranecz et al., 2011; Gehringer et al., 2013). Classically known, but also previously rather neglected animals like foxes could be an indicator for the prevalence of the pathogen (Kuehn et al., 2013).

The conclusion from these observations is that *Francisella* is widely present in the environment in Germany and that a wide range of wild animals (such as hares and wild boars), but also vectors (e.g., ticks as illustrated in the previous chapter) can be sources of infections in humans.

More research is needed to understand better the circumstances and mechanisms of a successful transmission of the pathogen from animal to animal and to humans. The main animal reservoir allowing the persistence and survival of *Francisella* in the wild is still unknown. Further research on this question is required. It would also be important to study changes in the ecosystems including populations of rodents and lagomorphs to understand better the impact of such changes on the prevalence of the pathogens in the wild and the occurrence of tularemia in humans.

## Diagnoses and clinical aspects of tularemia

*F. tularensis* ssp. *holarctica* causes usually a relatively mild form of tularemia in humans. The clinical manifestation of tularemia depends on the entry route of the bacterium into the organism and is defined by ulceroglandular or glandular form, oropharyngeal form, ocularglandular form and respiratory form (WHO Guidelines on Tularaemia, 2007)^1^. The primary common symptoms are fever and enlarged lymph nodes. The mean incubation time is 3–5 days with a range of 1–21 days. In the case of complications like suppuration, pneumonia and meningitis convalescence is often extended.

Among notified cases in Germany, the most frequent clinical presentations were glandular and ulceroglandular tularemia (Table 1). 7% of the patients presented with mixed forms and 14% could not be assigned (they typically only presented with fever (+ symptoms less typical for tularemia)). The latter could also represent typhoidal tularemia. Not all authors differentiate between “intestinal” and “oropharyngeal” forms of tularemia (WHO Guidelines on Tularaemia, 2007)^1^. When symptoms of both forms were present, these are listed under “combination” in Table 1. Collecting additional clinical details during routing surveillance could be considered to allow for a more accurate classification of cases.

**Table 1:** Notified tularemia cases with laboratory confirmation by clinical presentation, Germany, 2002–2016 (*n* = 257).

**Form**	***N***	**%**
Glandular (lymphadenitis and not meeting criteria for other forms)	81	32
Ulceroglandular (lymphadenitis + skin ulcer)	46	18
Pneumonic (dyspnea or pneumonia)	29	11
Intestinal (diarrhea, vomiting or abdominal pain)	22	9
Oropharyngeal (lymphadenitis + tonsillitis, pharyngitis, stomatitis)	18	7
Oculoglandular (lymphadenitis + conjunctivitis)	3	1
Combination (meeting criteria of >1 form)	19	7
other (symptoms not meeting any of the above criteria, e.g., “only fever”)	39	15
Total	257	100

Of the 257 cases of tularemia reported in 2002–2016, 39 (15.2%) were confirmed using an antigen assay, 175 (68.1%) serologically, 35 (13.6%) by culture and 58 (22.6%) by PCR (some cases were confirmed by a combination of methods). Median time from the onset of symptoms until notification (which typically occurs within 2 days of diagnosis) was 32 days (inter quartile range: 20–56 days), indicating that diagnosis is often delayed.

The laboratory diagnosis is often based on the detection of specific serum antibodies and/or of the *F. tularensis* DNA in clinical samples, but also in fixed and paraffin-embedded samples usually available from the pathology (own experience) (Grunow et al., 2000, 2001, 2014; Schmitt et al., 2005; Splettstoesser et al., 2005; Svensson et al., 2009b; Vogler et al., 2009b; Seibold et al., 2010; Jacob et al., 2011; Euler et al., 2012; Georgi et al., 2012; Sting et al., 2013; Chaignat et al., 2014; Becker et al., 2016; Challacombe et al., 2017). In addition, the isolation of the pathogen should be aimed at during an early stage of the disease for antibiotic sensitivity testing and further molecular-epidemiological investigation. The isolation of the pathogen was rarely successful when samples were obtained after initiation of an effective antibiotic treatment. On the other hand, we have seen that blood cultures taken early in the clinical course can lead to a successful isolation of the bacteria. Importantly, in the case of positive blood cultures with gram-negative bacteria, highly pathogenic bacterial organisms with extremely low infectious doses like *Brucella* or *Francisella* should be taken into consideration by the laboratory personnel and all following procedures should be carried out under appropriate biosafety conditions corresponding to BSL2 or BSL3 requirements (Directive 2000/54/EC http://eur-lex.europa.eu/legal-content/EN/TXT/?uri=CELEX:32000L0054). It should be noted that no natural resistances against first-line antibiotics have been described so far (Kudelina, 1973; Tomaso et al., 2005, 2017; Georgi et al., 2012; Karlsson et al., 2016). In Germany, the “Working Group of Competence and Treatment Centers for highly contagious and life-threatening diseases (STAKOB)” has developed recommendations for an adequate treatment of tularemia (http://www.rki.de/DE/Content/Kommissionen/Stakob/Stellungnahmen/Stellungnahme_Tularaemie.pdf?__blob=publicationFile).

Interestingly, we and others have diagnosed several tularemia patients with or without serious complications but with a prolonged time of recovery or even a recurrence of the disease after apparently adequate antibiotic treatment (Boone et al., 2015) (see case series below).

Case 1: We have reported a tularemia case in Germany who experienced a protracted clinical course over about 10 months (Grunow et al., 2015). The patient, a 22-year-old female, was living in an area in Germany where human cases of tularemia have been detected previously. The clinical history indicated a tick bite on the leg in June 2014. No further clinical signs occurred. In August 2014, the patient visited Turkey without any hints for an exposure risk. In mid-October, the patient became ill with fever and she also noted a vesiculo-papular rash. After treatment with ciprofloxacin over 5 days due to a simultaneous urinary tract infection, the patient recovered and was well. Early in December 2014, the patient experienced a unilateral cervical lymph node enlargement without other clinical symptoms and risk for exposure to *Francisella tularensis*. In mid-January 2015, the patient was hospitalized for further investigation. The Magnetic resonance imaging revealed an inflammatory liquefying lymph node on the right side and a lymphadenitis on the left sight of the cervix. A lymph node extirpation was conducted and the biopsy of the enlarged lymph node revealed an abscess-forming granulomatous inflammation with giant cells. A tissue sample was tested positive for *F. tularensis* by 16S-rPCR and the markers *tul4* and *fopA* confirmed *F. tularensis*. The RD1-PCR identified subspecies *holarctica*. Isolation of the living pathogen was not successful. The patient was empirically treated with cefuroxime and metronidazole. She remained sub-febrile (37.3°C). After the diagnosis of tularemia the treatment was switched to oral doxycycline but the temperature did not decline. A further treatment with ciprofloxacin p.o. over 14 d cured the sub-febrile temperature. In February 2015, the patient was in good general condition without fever, but still showed unilaterally enlarged lymph nodes and thickening of the cervical muscles, both painful on palpation. The site of the lymph node extirpation showed signs of delayed healing with secretion of a clear fluid (negative for *F. tularensis*). The vesiculo-papular exanthema in the cervical region and in the face was still visible. The serology at this time revealed an anti-*Francisella* titer of >100,000 by ELISA and a positive Western blot. After several ambulant controls, the patient was again hospitalized in March 2015 for further treatment with intravenous ciprofloxacin over 4 days and, after discharge from the hospital, further treatment with oral ciprofloxacin for an additional 11 days. In April 2015, out-patient control revealed secondary wound healing, good general condition, no fever and no enlarged lymph nodes.

Case 2: Another 68-year-old patient was described by H.L. Stich with a probable tick bite without cutaneous efflorescence and a time to diagnosis of about 8 weeks (Robert Koch-Institut, 2015). Because of a previously known neurological symptomatology, a neuro-somatic reason for the initial fever was assumed. After exclusion of this assumption, a detailed anamnesis including occupational information revealed that the patient had been a farmer for years and was also a hunter. This led to the assumption of a zoonotic infectious disease. The only sign of an infection was an elevated C-reactive protein (29.1 mg/l). Brucellosis and Q-fever were serologically excluded. Only 3 weeks after the first hospitalization, *F. tularensis* was considered and a clearly elevated antibody titer was detected. After this diagnosis, a specific treatment with doxycycline was initiated. The patient was discharged from the hospital after 4 weeks of therapy. Furthermore, it was revealed that 1 year before, four field hares infected with *F. tularensis* had been detected. This case shows that the consideration of differential diagnoses of tularemia might be hampered by other dominant clinical manifestations. In the case of pulmonary tularemia, lung cancer or tuberculosis are often the first choice of diagnoses. However, in the case of unclear fever and anamnestic information pointing toward contact with wild animals, tularemia should always be taken into diagnostic consideration.

Case 3: We described another 20-year-old female patient with a tick bite in her right hand occurring 5 months before consultation, followed by fever, chills and regional painful axillary lymphadenopathy (Lübbert et al., 2009). Interestingly, the empiric antibiotic treatment with doxycycline and ciprofloxacin had led to defervescence but no change in painful lymph node swelling. Surgical removal of a cubital lymph node was performed 3 months after the tick bite. 5 months after the tick bite laboratory findings were normal except for moderate elevation of C-reactive protein. Detection of specific serum antibodies against *F. tularensis* confirmed the suspected clinical diagnosis of ulceroglandular tularemia. The pathogen could not be detected by isolation or PCR. The histology of the removed painful axillary lymph node showed reticulocytic, abscess-forming lymphadenitis with a pseudotuberculosis type of granulomatosis and negative acid-fast staining. A complete recovery was achieved without renewed antibiotic treatment.

In 2016/2017 we have seen several additional cases with some peculiarities. An outbreak of tularemia with 6 serologically confirmed cases among 30 mostly volunteering participants of a grape harvest in Rhineland-Palatinate which took place at the beginning of October 2016 was reported to ProMED (http://www.promedmail.org/post/4647937). 3–8 days after the activity, the affected individuals developed symptoms like high fevers, general malaise, and unilateral marked cervical lymphadenopathy suggesting an oropharyngeal route of infection. Interestingly, one patient showed symptoms of an oro-pharyngeal tularemia only 24 days after the exposure. Another patient experienced a severe clinical course of more than 2.5 months. Due to the absence of additional epidemiological hinds, the diagnosis of tularemia was delayed. Three of the six patients were briefly hospitalized. An epidemiological outbreak investigation was initiated considering a broad range of environmental exposures and food-stuffs consumed during the event; the results have not been published yet.

The conclusion from these case reports is that *F. tularensis* ssp. *holarctica* can cause long-lasting clinical courses of disease. Tularemia can also occur as a recurrent disease over several months. In addition, the onset of an oro-pharyngeal tularemia can occur as late as 3 weeks after a relevant exposure. The pathologic mechanisms of a protracted course of tularemia are still unclear but an immune reaction to persistent antigens or pathogens appears a plausible explanation (Straube and Hess, 1986; Hanke et al., 2009; Lübbert et al., 2009; Capka et al., 2010; Dlugaiczyk et al., 2010; Fritzsch and Splettstoesser, 2010; Hauri et al., 2010; Potz-Biedermann et al., 2011; Bulut et al., 2013; Weile et al., 2013; Kohlmann et al., 2014; Guerpillon et al., 2016). The infection route often remains unclear and can be suggested only by a detailed anamnestic investigation. In late stages of the disease, especially after appropriate antibiotic treatment, living *Francisella* cannot be detected, but PCR investigation of tissue material and serology confirms the disease. Awareness of clinicians should be raised to consider tularemia in patients with enlarged lymph nodes and fever and a relevant exposure. This is even more important when atypical or very seldom clinical manifestations are seen like in pneumonic tularemia with lung nodules suggestive of malignancy, manifestation with uveitis or endocarditis (Terrada et al., 2016; Gaci et al., 2017; Odegaard et al., 2017).

## One-health aspect of tularemia

Tularemia is a zoonotic disease. Cases of disease in humans and animals are notifiable in Germany and several other European countries. To understand the complexity of the disease, a close cooperation of veterinary medicine, human medicine, animal health and public health is crucial. Elevated risks of exposure or even outbreaks in humans are often preceded by observation of the disease in animals (Mörner, 1992; Splettstoesser et al., 2007, 2009; Gyuranecz et al., 2011; Grunow et al., 2012; Kuehn et al., 2013; Otto et al., 2014, 2015; Hestvik et al., 2015). Thus, a timely sharing of data concerning cases in animals with the public or the public health sector and awareness raising in outbreak situations could facilitate the early detection or prevention of cases in humans. The source of infection for human cases with tularemia often cannot be identified; however, in several cases, clues for an infection can be obtained through the anamnesis. In very rare cases the source of infection can be verified. If not eliminated, the sources of infection can pose a high risk to other people. This can include e.g., drinking water contaminated by dead animals, freshly pressed juice from field fruits, fresh or frozen stored meat from infected animals. Unless direct identification of the infectious sources is done, transmission of bacteria by aerosols and dust, e.g., when cleaning barns or cutting grass could become a continuing event. As insect vectors can also transmit *Francisella*, knowledge of the prevalence in ticks, mosquitoes and possibly other insects is of interest. The investigation into sources of infection in sporadic cases and outbreaks and also the investigation of the prevalence of the pathogen in animals and environmental samples require a strong one-health approach in which specialists of animal health and of human health work together. This requires staff and financial resources which are often scarce. Compared to other infectious diseases, tularemia is rare and the burden of disease is relatively low. Thus, funders and decision makers are hesitant to allocate resources required to understand better the epidemiology of this disease and to prevent further cases. While the disease is rare, surveillance of human cases indicates a re-emergence with an approximately ten-fold increase of notified cases in Germany in a period of 15 years. Possible reasons include an increased presence of the pathogen in the environment and more frequent contact between humans and wildlife through leisure activities. However, an increased awareness of the disease and more frequent testing might have contributed to the continuous rise of notified cases.

Interactions between pathogen, the environment, its hosts and humans, render this interesting and potentially serious infectious disease a complex topic. In case of rare diseases like tularemia, utilizing international synergies, data, and expertise, ideally in internationally sponsored research projects, seems important to further advance prevention and control.

## Author contributions

MF analyzed and described the surveillance data. RG is the head of the German Consultant Laboratory for Tularemia and in this function conducted laboratory diagnostics and epidemiological investigations and observed clinical cases which were summarized here together with the literature review. MF, KH, DJ, and RG drafted the manuscript. All authors have critically revised the manuscript.

### Conflict of interest statement

The authors declare that the research was conducted in the absence of any commercial or financial relationships that could be construed as a potential conflict of interest.

## Footnotes

^1^http://apps.who.int/iris/bitstream/10665/43793/1/9789241547376_eng.pdf, last check February 2018.
